# Effects of Access Cavity Design and Placement Techniques on Mineral Trioxide Aggregate Obturation Quality in Simulated Immature Teeth: A Micro-Computed Tomography Study

**DOI:** 10.3390/medicina60060878

**Published:** 2024-05-27

**Authors:** Emine Odabaşı Tezer, Arda Buyuksungur, Berkan Celikten, Pınar Hava Dursun, Fatma Semra Sevimay

**Affiliations:** Department of Endodontics, Faculty of Dentistry, Ankara University, Ankara 06560, Turkey; abuyuksungur@ankara.edu.tr (A.B.); berkancelikten@gmail.com (B.C.); phdursun@ankara.edu.tr (P.H.D.); ssevimay@dentistry.ankara.edu.tr (F.S.S.)

**Keywords:** micro-computed tomography, minimal access cavity, MTA apical plug, obturation quality, placement techniques, XP-endo Shaper

## Abstract

*Background and Objectives:* In teeth with open apices, performing single session apexification is a challenging treatment due to the difficulty in handling mineral trioxide aggregate (MTA). Minimally invasive approaches in dentistry have also influenced the cavity designs in endodontics. Until now, different techniques have not been investigated in addition to manual condensation during the process of placing MTA in traditional (TradACs) or conservative (ConsACs) endodontic access cavities. The aim of this in vitro study was to compare and evaluate the obturation quality of MTA apical plugs placed with different techniques in TradACs or ConsACs. *Materials and Methods*: Sixty upper central teeth were divided into two main groups based on cavity design, and then each main group was further divided into three subgroups according to MTA placement techniques (*n* = 10): TradAC-manual, TradAC-manual + indirect ultrasonic activation, TradAC-manual + XP-endo Shaper (XPS), ConsAC-manual, ConsAC-manual + indirect ultrasonic activation, and ConsAC-manual + XPS. Subsequently, the porosity percentages in the MTA apical plug were analyzed using micro-computed tomography. The statistical analysis was performed using the Kruskal–Wallis H test and Mann–Whitney *U* test. Statistical significance was set at *p* < 0.05. *Results:* There were differences in volume of porosity percentages (%) according to cavity designs and MTA application techniques (*p* < 0.05). Except for the XPS group, more porosity was observed in ConsACs compared to TradACs. In TradACs, the significantly lowest open and total porosity was observed in the manual, ultrasonic, and XPS techniques, respectively. In ConsACs, the significantly lowest porosity was observed in the manual, XPS, and ultrasonic techniques, respectively (*p <* 0.05). *Conclusions:* In MTA obturation, cavity designs and application techniques had an impact on the MTA porosity. Creating an apical plug in ConsACs may result in more porosity compared to TradACs, especially when manual or indirect ultrasonic activation is preferred. Opting for the manual technique alone may be considered sufficient for controlling porosity for both TradACs and ConsACs.

## 1. Introduction

Dental trauma or caries can result in pulpal necrosis and apical periodontitis in immature permanent teeth [1]. Many patients in childhood lose their teeth due to this reason [2]. The thin apical root canal dentin walls, short roots, and increased crown–root ratio in necrotic immature teeth adversely affect the treatment prognosis [3]. Performing non-surgical conventional root canal treatment in such teeth jeopardizes long-term success due to the risk of root canal filling material extrusion into the periodontium caused by the loss of apical constriction, difficulty in achieving apical seal, and incomplete drying of the root canal system due to moisture from the periapical region [4].

### 1.1. Necrotic Immature Permanent Teeth and Endodontic Approaches

Recommended endodontic treatment options for immature necrotic teeth include apexification, pulp revascularization/revitalization, and regenerative endodontics. Apexification is the encouragement of apical calcified barrier formation by obturating the root canal of an immature tooth with mineral trioxide aggregate (MTA) or calcium hydroxide [Ca(OH)_2_]. In revascularization/revitalization or regenerative endodontics, in essence, the root canal system is disinfected with various antimicrobial or antibiotic agents, and bleeding is induced into the root canal through the apical foramen to promote the establishment of tissue that will continue root development.

### 1.2. Apexification with Calcium Hydroxide or Mineral Trioxide Aggregate

Completing root mineralization is critical, especially in young permanent teeth with thin root canal dentin walls, to maintain tooth fracture resistance. Therefore, regenerative endodontics is particularly suitable for patients aged 6–17 years old with fragile root dentin walls, no crown–root fractures, an apical opening diameter of more than 1.1 mm, and no systemic health issues [2]. However, in teeth that are already necrotic or progressing from irreversible pulpitis towards necrosis, and have adequate thickness of root dentin walls, apexification treatments are more suitable than regenerative endodontics [5]. Therefore, apexification emerges as the preferred optimal treatment option when there is sufficient presence of root canal dentin walls to support the tooth’s fracture resistance [2]. Apexification involves inducing the development of hard tissue, which contributes to the prognosis of the tooth, by placing Ca(OH)_2_, MTA, or bioceramics in an immature permanent root canal after effective chemo-mechanical preparation [5]. A meta-analysis that included randomized controlled trials reported similar success rates among the three materials in terms of clinical, radiographic, and apical plug formation [5]. However, apexification with Ca(OH)_2_ is a long-term process that requires patient compliance. In this approach, an ideal environment for supporting apical calcification is provided by ensuring disinfection of the root canal system with Ca(OH)_2_, which is usually replaced in monthly appointments. The occurrence of apical closure is confirmed with periodic periapical radiographs [6]. At the same time, apexification with Ca(OH)_2_ can lead to structural changes in dentin, reducing the tooth’s fracture resistance and potentially leading to reinfection in teeth with long-term temporary obturation [7]. Due to these disadvantages, in recent years, MTA has been preferred instead of Ca(OH)_2_ in apexification treatment as it can be applied as an apical obturation material in a single session [8]. Various bioceramics can also be used in apexification therapy instead of MTA to overcome its drawbacks, like tooth discoloration, long setting time, and difficult manipulation, ensuring effective sealing at the apex [9,10]. MTA can induce hard tissue formation [11] and regress apical periodontitis due to its successful apical seal in necrotic and immature teeth [12]. Therefore, the MTA apical plug is a reliable treatment option for teeth with an open apex [13], and the survival rate is 97% [14].

### 1.3. Orthograde MTA Applications: Clinical Challenges and Limitations

Techniques during the preparation or application of the material used in dentistry may affect its performance and clinical effectiveness [15,16], insomuch that, as clinically, the correct and effective application of the material is more important than the properties it possesses [17]. It has been emphasized that the techniques preferred for effective placement of MTA are particularly important, especially in teeth with open apices [17,18]. In this regard, the orthograde MTA condensation technique presents certain challenges. Lack of direct vision, cautious application of condensation pressure due to the apical opening, and irregular dentin walls in open apices may prevent MTA to completely seal the dentin surface [17]. MTA is also highlighted as a material that is quite difficult to manipulate. When MTA powder and liquid are mixed, MTA transforms into a slurry paste, which poses challenges in its handling and compaction into the narrow and confined spaces of the root canal [19,20]. Moreover, the inadequacy of manipulation of the MTA prevents its effective condensation, resulting in the formation of porosities. While porosities facilitate water ingress for the hydration reaction that enables MTA to harden [21], there is a negative correlation between the total porosity of MTA and its flexural strength [22], which is an important indicator of the material’s clinical mechanical performance [23]. In a recent cross-sectional study, it was reported that teeth that received root canal treatment had apical periodontitis at a rate as high as 40%. Porosity directly influences the passage of bacteria and bacterial by-products into the periapical area [24]. Lower porosity in endodontic filling materials has been associated with reduced bacterial transmission [25]. In conclusion, achieving a hermetic obturation of the root canal system is crucial irrespective of the chosen material or technique for root canal filling. Therefore, evaluating the presence of porosities can be a criterion that provides insight into the quality of root canal obturation [26]. Considering these perspectives, the optimal method for the orthograde placement of MTA is an important issue due to its potential contribution to long-term outcomes and tooth survival rates for teeth with open apices.

### 1.4. MTA Application Techniques

To improve the obturation quality of MTA, approaches such as manual compaction with hand files [19], pluggers [27], lentulo [28], and MTA pellet blocks [29] have been investigated over many years. Despite the recommended techniques, difficulties persist in obturating MTA void-free due to various factors, such as operator skill, material choice, root canal geometry, setting time, and application techniques [30]. The use of ultrasonic activation in MTA obturation has been proposed to overcome this limitation [20,27,31,32,33]. However, to date, no consensus has been reached regarding the ideal technique to be preferred when creating an apical plug with MTA [34] and the need for research on determining the optimal technique for MTA obturation has been emphasized [20]. Recently, the quality of MTA obturation has also been evaluated using rotary instruments [35]. XP-endo Shaper (XPS) (FKG Dentaire SA, La Chaux-de-Fonds, Switzerland) is a recently introduced single file root canal preparation system manufactured with M-wire technology. Therefore, in this study, the effects of XPS’s characteristic three-dimensional movement and indirect ultrasonic activation were evaluated.

### 1.5. Minimal Endodontic Access Cavities

One of the risks threatening the prognosis of teeth that have undergone root canal treatment is the amount of remaining tooth structure, which can be as critical as reinfection. In this context, the concepts of extension for prevention or convenience form are being questioned to optimize the outcomes of endodontic treatment. Minimally invasive endodontics is based on the principle of preserving biological tissues as much as possible while achieving the goals of endodontic treatment. In traditional endodontic access cavities, a geometric form is established that directly accesses the root canal orifices, potentially reducing the tooth’s fracture strength [36]. On the other hand, modern endodontic tools allow for the creation of minimally invasive endodontic access cavities, preserving enamel and dentin to the maximum extent [37]. Minimally invasive endodontics prioritizes the protection of remaining tooth tissue, encompassing various applications from access cavity design to root canal preparation and even regenerative endodontics [38]. The effects of minimally invasive endodontic cavities have been extensively studied across numerous endodontic topics, such as orifice location, mechanical preparation, instrument fracture, root canal disinfection, root canal filling, and retreatment [39]. Minimal invasive approaches should be tested not only in the mentioned topics but across all existing endodontic approaches [40]. However, to date, no studies in the literature have addressed the creation of an apical plug in conservative access cavities. In this regard, clinicians may encounter situations where they prefer minimal cavity design for creating an MTA apical plug due to various reasons. Additionally, the effect of different application techniques on the quality of MTA obturation in traditional and conservative endodontic access cavities has not been investigated. Thus, the focus of this study was to test the null hypothesis of whether the application technique or cavity design would not have an effect on MTA obturation quality. 

### 1.6. Aim of the Study

To our knowledge, no study exists examining the obturation quality of MTA apical plugs with different placement techniques in different access cavities. Therefore, the aim of this in vitro study was to evaluate and compare the quality of MTA obturated using manual, indirect ultrasonic activation, and rotary delivery (XP-endo Shaper, XPS, FKG Dentaire SA, La Chaux-de-Fonds, Switzerland) techniques in conservative (ConsACs) or traditional endodontic access cavities (TradACs) in simulated immature teeth with micro-computed tomography (micro-CT).

## 2. Materials and Methods

### 2.1. Ethical Approval and Sample Size Calculation

This study adhered to the Declaration of Helsinki and received approval from the ethics committee of the Ankara University Faculty of Dentistry (registration number: 36290600/77). The sample size of this in vitro study was calculated using the G*Power statistical program (ver. 3.1.9.7). The test power was calculated at 0.80 with an effect size of 0.5 and a type-1 error (α) set at 0.05. The total sample size was then calculated as 60, with 10 samples (*n* = 10) in each group. In this case, the actual power value was found to be 83%. 

### 2.2. Selection and Preparation of Samples

Extracted teeth were collected from the Ankara University Faculty of Dentistry Periodontology and Oral and Maxillofacial Surgery departments as a result of extraction indications for reasons unrelated to this study. Sixty intact, freshly extracted upper central human teeth were used in the study. Once the teeth were extracted, any soft and hard tissue remnants on them were removed with a periodontal curette. Subsequently, the teeth were disinfected by scrubbing with 5% sodium hypochlorite (NaOCl) to remove organic residues soaked in a gauze pad and then rinsed under running water. Periapical radiographs (Gendex Dental Systems, Hatfield, PA, USA) were taken in mesio-distal and bucco-lingual directions to evaluate the presence of resorption and calcifications in the samples. The teeth that met the inclusion and exclusion criteria were selected (Table 1). Then, the teeth were stored in a light-proof jar filled with distilled water containing 0.1% thymol crystals at room temperature for approximately 3 months, which encompassed the time until the required number of teeth for the experiment was obtained and throughout the duration of the experiment for approximately 3 months. 

### 2.3. Simulation of Periodontium

The root tips were removed using a diamond disc (109-035C, Midwest Once^®^ Diamond Burs, Dentsply Sirona, Charlotte, NC, USA) with water cooling, leaving a working length of 18 mm from the reference point at the midpoint of the incisal edge to the apex. The periodontal ligament model was performed as described by Ghasemi et al. [41]. Firstly, the roots were covered with melted preparation wax (Bego, Bremen, Germany) to approximately 0.2–0.3 mm thickness to simulate the periodontium up to the cemento-enamel junction. Then, plastic molds with dimensions of 20 mm × 20 mm × 18 mm were filled with gypsum (plaster of Paris). Finally, the teeth were embedded so that the long axis of the tooth was perpendicular to the horizontal axis of the gypsum block up to the cemento-enamel junction. After the gypsum had completely set in 1 h, the molds were immersed in a container filled with hot water to melt the preparation wax. After the wax was melted, the teeth were removed from the gypsum block. The excess wax remaining on the root surfaces or in the artificial gypsum socket was removed with a carver. Polyethylene impression material (3M ESPE TM Deutschland, GmbH, Seefeld, Germany) was then poured into the artificial socket using a cement spatula, and the samples were repositioned.

**Figure 1:**
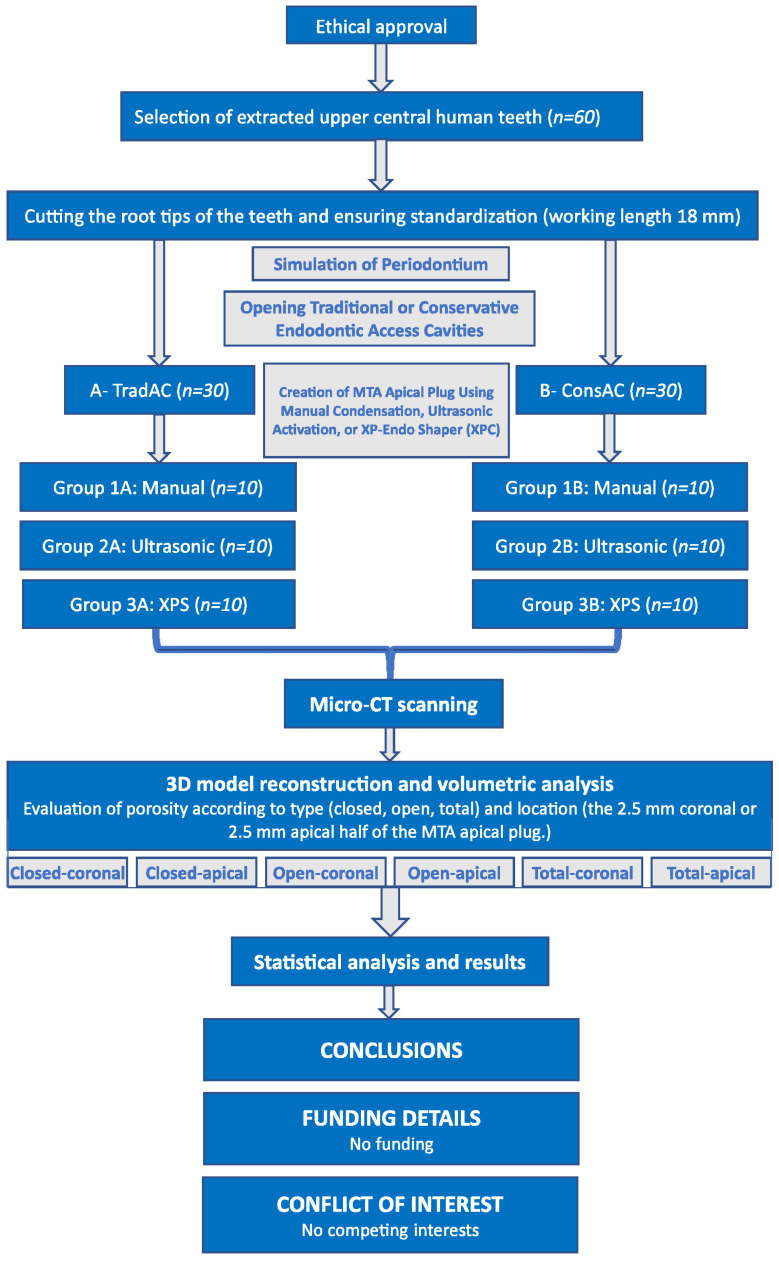
Flowchart showing the study design.

**Figure 2:**
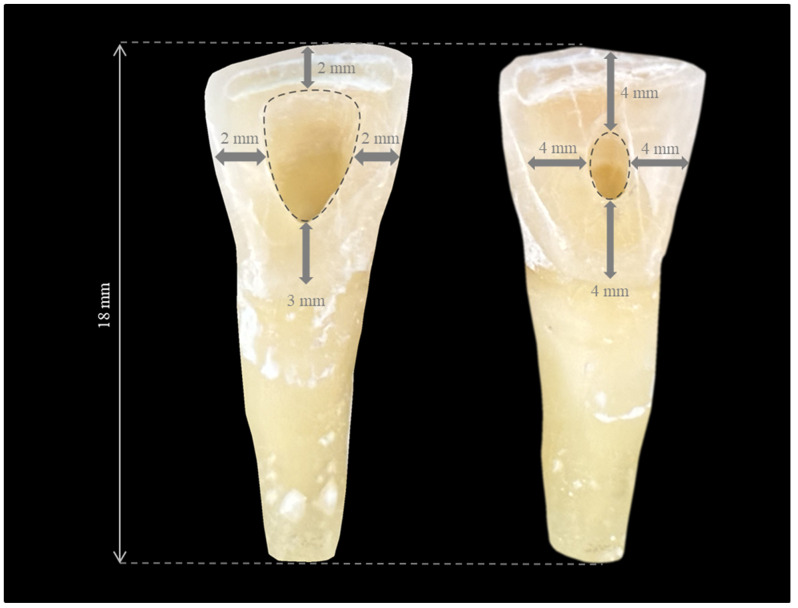
Traditional endodontic access cavity TradAC (**left**) and conservative endodontic access cavity (MinAC) (**right**) with open apex simulation.

A flowchart summarizing the entire study protocol is presented in Figure 1. Samples were randomly divided into two main groups, and similarly-sized traditional endodontic access cavities (TradACs) or conservative endodontic access cavities (ConsACs) were opened using a diamond access bur (Lexicon, Dentsply-Maillefer, Baillagues, Switzerland) numbered #4 and #2, respectively, using a dental operating microscope (×6, Leica, M320, Singapore), as described by Jurado et al. (Figure 2) [42]. 

### 2.4. Opening Traditional Access Cavities (TradACs)

In TradACs, markings were made with a pencil on the palatal surface of the teeth 3 mm above the cemento-enamel junction, 2 mm below the incisal edge, and 2 mm medial to both the mesial and distal edges. Cavity dimensions were determined adhering to these boundaries. A #4 diamond access bur (Lexicon, Dentsply Maillefer, Baillagues, Switzerland) was initially positioned perpendicular to the palatal surface, then maintained parallel to the long axis of the tooth to reach the pulp chamber under water cooling. TradACs were completed by removing the pulp roof and horns, forming a rounded triangle shape with divergent angled walls. 

### 2.5. Opening Conservative Access Cavities (ConsACs)

In ConsACs, markings were made with a pencil on the palatal surface of the teeth 4 mm above the cemento-enamel junction, 4 mm below the incisal edge, and 4 mm medial to both the mesial and distal edges. Cavity dimensions were determined adhering to these boundaries. A #2 diamond access bur (Lexicon, Dentsply Maillefer, Baillagues, Switzerland) was initially positioned perpendicular to the palatal surface, then maintained parallel to the long axis of the tooth to reach the pulp chamber under water cooling. Finally, ConsACs were completed in the form of an ovoid, leaving the pulp horns intact and preserving the pericervical dentin. 

The apical opening was checked with a #20 K-file (Dentsply Sirona, Ballaigues, Switzerland). Mechanical preparation was performed with ProTaper Next (Dentsply Maillefer, Ballaigues, Switzerland) up to #F5 (50/.06) using the X-Smart Plus endomotor (Dentsply Maillefer, Ballaigues, Switzerland) with an orthograde approach in all samples. The root canals were irrigated with 2 mL of 5% NaOCl (Werax, Izmir, Turkey) at each file change using a 27-gauge irrigation needle (NaviTip; Ultradent, South Jordan, UT, USA). Then, samples were removed from the socket. ProTaper Next #F4 (40/.06; Dentsply Maillefer, Switzerland) was used retrogradely with a 16 mm working length to create the apical bell-shaped opening [34]. The samples were reinserted into the socket after completing apical shaping.

Final irrigation was performed with 5 mL of 5% NaOCl, 5 mL of 17% Ethylenediaminetetraacetic acid (EDTA) (Werax, Izmir, Turkey) and distilled water for 1 min, respectively. The root canals were dried with paper point cones (Diadent, Seoul, Republic of Korea). Then, the teeth were divided into six groups with 2 main groups according to the endodontic access cavities (TradACs and ConsACs) and 3 subgroups according to the MTA application techniques (manual condensation (manual), manual condensation + indirect ultrasonic activation (ultrasonic), manual condensation + XP-endo Shaper (XPS) [FKG Dentaire SA, La Chaux-de-Fonds, Switzerland]) in each main group (*n* = 10), randomly (www.random.org, accessed on 5 August 2023). The samples were kept in water at 37 °C, creating an environment similar to clinical conditions. MTA (Angelus, Soluçoes Odontologicas, Londrina, Brazil) was mixed with distilled water on a paper pad, using a water-to-powder ratio of 1:3 until it reached a homogeneous wet sand consistency, and placed into the canals with an MTA gun (Dentsply Maillefer, Switzerland) in all groups. Then, it was condensed with an endodontic plugger (35/.03, Fanta Dental, Shanghai, China) in layers of 2 mm, 2 mm, and 1 mm for a total of 5 mm thickness. MTA condensation was completed after each placement layer by following the procedures below according to the subgroups (*n* = 10): 

Group 1—manual condensation: MTA was manually condensed with an endodontic plugger. 

Group 2—indirect ultrasonic activation: After each manual placement layer mentioned above, indirect ultrasonic activation was applied to the MTA. The ultrasonic tip CPR-1 (Dentsply Maillefer) attached to the piezoelectric ultrasonic unit (EMS Piezon Master 400; Electro, Medical Systems SA, Nyon, Switzerland) set at 25 kHz was activated for 10 s by touching the shaft of the plugger. 

Group 3—XP-endo Shaper: The condensation of MTA was completed using the XP-endo Shaper (XPS) (FKG Dentaire SA, La Chaux-de-Fonds, Switzerland) after each manual placement layer mentioned above. The XPS file was inserted into the canal 1 mm shorter than the working length (17 mm) at 800 rpm in a counterclockwise direction. Upon reaching the predetermined working length, it was gently withdrawn from the canal while the file was still in rotary motion. This procedure was repeated three times. 

A single endodontist with 10 years of experience performed all experiment steps under an operating microscope (x16, Leica, M320, Singapore). Voids, adaptation, and thickness of MTA were confirmed by digital periapical radiographs. After placing a moist cotton pellet into the pulp chamber, temporary filling (Coltosol F, Coltène/Whaledent AG, Altstätten, Switzerland) was used to close the access cavities. The samples were kept in an incubator at 37 °C for 7 days in 100% humidity.

### 2.6. Micro-CT Scanning and Analyzing

Subsequently, the samples were scanned with 80 kV and 125 mA using a desktop micro-CT scanner, 1275 micro-CT (Bruker-microCT, Kontich, Belgium) with a pixel size of 15 μm. The procedure was performed using a 1 mm aluminum filter and the scan was conducted 360° with a 0.2 rotation step and 50 ms exposure time. The teeth were placed into orthodontic wax to block any movement. NReceon software (v. 1.7.4.2, Bruker-microCT, Kontich, Belgium) was used for the reconstruction of the raw data. Images were reconstructed with the parameters ring artifact reduction factor 7, smoothing 3, and beam hardening correction 38% to decrease any artifact. CTAn software (v. 1.23.0., Bruker micro-CT, Kontich, Belgium) was used for the analysis of the images and quantitative evaluation of the MTA volume, porosity, and the other analysis. ROI (region of interest) was selected to include the root canal for the calculation of porosity and analysis. Then, the analysis was carried out with the apical region of the teeth (5 mm) and the volumes of interest (VOIs) were created, and the analysis was carried out using VOIs. 

The analyses were carried out using segmentation within the ROIs and VOIs. Grayscale thresholds were used as global thresholding. Different segmentation was used for the separation of the MTA and pores. After thresholding, binarization was used for the quantitative analyses.

Images were observed in 3D with CTVOX 3.30.r1403 version. 2D analyses were performed with Dataviewer software 1.5.6.2. All porosity analyses were carried out with CTAn software. Then, the 2.5 mm coronal and 2.5 mm apical part of the MTA apical plug was evaluated separately and typed as closed, open, and total porosity. If the voids were within the MTA, it was defined as closed porosity, and if the voids were between the dentin wall and the MTA, it was defined as open porosity. The sum of open and closed porosity was called total porosity. Therefore, MTA plug porosity is expressed as follows: closed-coronal, closed-apical, open-coronal, open-apical, total-coronal, and total-apical porosity. Micro-CT analysis was blindly carried out by an operator with 13 years of experience.

### 2.7. Statistical Analysis

Statistical analysis was conducted using SPSS (IBM SPSS for Windows, ver.26). The normality of continuous variables, such as the volume of porosity percentages (%), was evaluated using the Shapiro–Wilk test. The Shapiro–Wilk test results indicated non-normal distribution of the dataset. It was determined that continuous variables did not follow a normal distribution. Therefore, median, interquartile range (IQR) values were used in the representation of descriptive statistics. Given the non-normal distribution of continuous variables, non-parametric tests were employed for statistical analysis. The Mann–Whitney *U* test was used for comparisons between two groups, and the Kruskall–Wallis H Test was used for comparisons among three or more groups. Following this analysis, the post hoc Bonferroni (multiple) comparison test was used for groups with significant differences. The data were evaluated using descriptive statistical methods. A significance level of 0.05 was determined. 

## 3. Results

**Figure 3:**
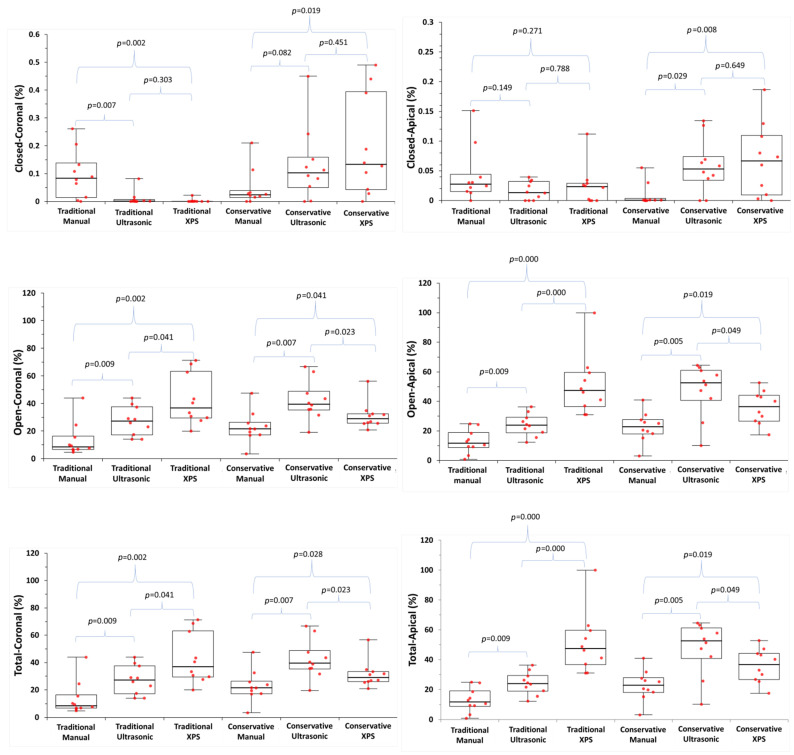
Closed-coronal, closed-apical, open-coronal, open-apical, total-coronal, and total-apical porosity percentages (%) according to the cavity designs (traditional or conservative) and application techniques (manual condensation (manual), manual condensation + indirect ultrasonic activation (ultrasonic), manual condensation + XP-endo Shaper (XPS) [FKG Dentaire SA, La Chaux-de-Fonds, Switzerland]). Box-and-whisker plots with the median (horizontal line), interquartile range (box) and outlier (circles) values are shown. *p* < 0.05 is statistically significant.

**Figure 4:**
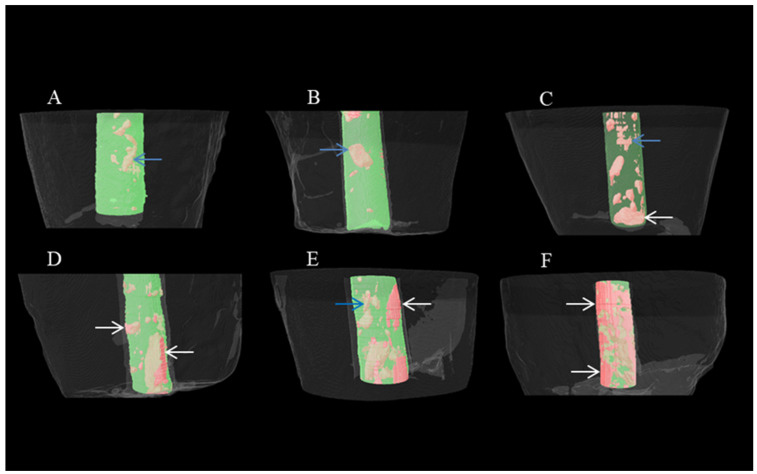
Representative micro-computed tomography (micro-CT) images showing porosities in MTA apical plugs based on experimental groups: (**A**)—TradAC-manual, (**B**)—ConsAC-manual, (**C**)—TradAC-ultrasonic, (**D**)—ConsAC-XPS, (**E**)—TradAC-XPS, (**F**)—ConsAC-ultrasonic (the blue arrow indicates closed porosity, the white arrow indicates open porosity).

The median and interquartile porosity percentage values for the application techniques in the ConsAC and TradAC groups are presented in Table 2. Median, interquartile range, and outlier values are shown in Figure 3. Representative micro-CT images depicting each group are shown in Figure 4.

There are significant differences in porosity percentages among MTA application techniques in both TradACs and ConsACs (*p* < 0.05). In TradACs, open and total porosity percentages were lower compared to ConsACs, except in the XPS group (*p* < 0.05). In TradACs, open and total porosity were lowest in the manual, ultrasonic, and XPS groups, respectively, while in ConsACs, they were lowest in the manual, XPS, and ultrasonic groups (*p* < 0.05). Open and total porosity in the XPS group were lower in ConsACs compared to TradACs (*p* < 0.05). In both TradACs and ConsACs, the manual technique resulted in less open and total porosity compared to ultrasonic and XPS (*p* < 0.05).

## 4. Discussion

The literature presents a fragmented view on the optimal technique for MTA placement, with various studies advocating for manual, ultrasonic, or rotary methods without reaching a consensus [35,43,44]. Furthermore, the influence of access cavity design—conservative versus traditional—on the quality of MTA obturation remains underexplored. This study aims to address this gap by systematically evaluating the impact of different access cavity designs and placement techniques on the obturation quality of MTA apical plugs in simulated immature teeth, utilizing micro-CT detailed analysis. Such an investigation could be crucial for advancing our understanding of optimal endodontic practices for treating immature necrotic teeth, ultimately enhancing treatment outcomes and tooth survival rates. 

In the present study, freshly extracted teeth that retained moisture in their structure and had not become brittle were used to prevent potential cracks and fractures on the root surface from affecting the experimental results. Extracted human teeth better reflected clinical conditions compared to artificial teeth. For standardization, teeth were included that had similar dimensions, canal diameters, and canal anatomies, as well as that were entirely intact with no compromise in integrity for any reason. Also, the working length was standardized to 18 mm during the open apex simulation. A study conducted in the Turkish population reported that upper central incisors exhibited 100% type I canal configuration according to the Vertucci classification, indicating a single root and single canal [45]. Additionally, the frequency of dental trauma injuries reported during childhood and adolescence varies between 4–59%, often affecting the upper central incisors and frequently resulting in the cessation of root development and pulp necrosis [46]. Due to the challenges of the MTA in adapting to the irregularities in complex canal structures, caution is advised. To mitigate this concern, upper central incisors were chosen for their minimal variation in root canal configuration and high susceptibility to dental trauma in immature teeth. Since extracted human teeth can contribute to cross-contamination, they were immediately immersed in distilled water containing 0.1% thymol crystals after extraction for antimicrobial control [47]. They were kept in this solution until the experiment was completed. 

The use of dental operative microscopes has led to notable improvements in the effectiveness of dental treatments. Non-surgical and surgical endodontics necessitate varying magnification levels. These can be categorized into low (×2–×8), medium (×8–×16), and high (×16–×25) magnification. Medium magnification offers depth of field and brightness, making it suitable for many clinical situations such as orifice location, apical resection, root surface analysis, fracture diagnosis, and obturation [48]. Therefore, in this study, optimal view in the apical third, which is the deepest part of the root, was achieved with a magnification of ×16 providing depth of focus and illumination.

In this study, quantitative data were acquired through micro-CT analysis, which offers a close representation of the clinical scenario without causing any destructive alterations to the samples during examination [49]. Light microscopy and laser confocal scanning microscopy are useful for detecting voids between the material and dentin, but they do not have the required resolution to measure fine interfacial gaps [50]. Despite providing high resolution, sections obtained for evaluating interfacial surfaces and marginal adaptation of materials in scanning electron microscopy (SEM) can create unwanted voids and lead to misleading results [51]. Therefore, the ability to repeatedly examine the image without any alteration to the sample, provided by micro-CT scanning, is a significant advantage. The extensive number of cross-sectional images obtained from the samples allows for a very detailed evaluation, both quantitatively and qualitatively [49]. Additionally, the three-dimensional image of the scanned object can be obtained and recorded. If desired, the data can be compared with biological, histological, and mechanical test results. Micro-CT provides three-dimensional analysis for detecting open and closed porosities, determining their ratio, surface area, volume, and localization in porous materials [52]. In previous studies, micro-CT has been used to evaluate the porosity within MTA [20,26,34,35,53]. Therefore, in the current study, micro-CT scans and analyses were used to determine the volume of porosity percentages.

The concept of entirely filling the root canal with MTA is based on its potential to increase the tooth’s fracture resistance, prevent bacterial leakage, and neutralize bacteria [19]. However, Jho et al. [54] argued against the full obturation of the root canal system with MTA because of the lower filling quality. Other significant limitations of total MTA filling include the considerable difficulty in its removal when necessary. At the same time, even white MTA can potentially cause tooth discoloration. In a study comparing the sealability of MTA samples with thicknesses of 3 mm, 5 mm, or the entire root filled with MTA, no significant difference was found between the groups four weeks later [30]. During a 70-day test period in a model with *Actinomyces viscosus*, a 5 mm MTA apical plug demonstrated complete resistance to microbial leakage [55]. Moreover, the 5 mm thickness exhibited notably higher microhardness compared to the 2 mm thickness [56] and can completely remove bacteria [57]. For this reason, the MTA apical plug was placed with a thickness of 5 mm, as it is more predictable clinically. Decoronization was not preferred to mimic the clinical situation, as the natural position of the incisal edge would change, and also to avoid affecting the cavity design. This is also one of the limitations of the study due to causing standardization bias.

In this study, the obturation quality of MTA was evaluated by calculating the open, closed, and total volume of porosity percentages. Open porosity is a void at the interface between the root canal dentin wall and MTA, which can create a space for the passage and proliferation of microorganisms. Closed porosity refers to the voids within MTA itself and generally has less clinical significance compared to open porosity. Total porosity is the sum of open and closed porosities. Significant differences in porosity percentages were observed according to cavity designs and application techniques. Therefore, the null hypothesis of the study was rejected.

Some studies have reported that the presence of gaps in root canal filling has no significant impact on clinical success alone, regardless of evaluation criteria [58,59]. Conversely, open porosity, influenced by the gap at the dentin–material interface [34], is regarded as a criterion for material adaptation [60]. Moreover, Dioguardi et al. [61] claimed that insufficient root canal obturation in the apical or coronal region results in reinfection. Thus, it was important to analyze the apico-coronally distributed pattern of porosities, which may have different meanings in clinical terms. 

In the current study, MTA application techniques and cavity designs influenced MTA porosity. The porosity percentages in the TradACs were significantly lower compared to the ConsACs in the manual and ultrasonic groups, except for the closed-coronal porosity percentages in the manual group. The convenience form of TradACs may have facilitated MTA plug manipulation, resulting in a less porous obturation compared to ConsACs. Straight entry into the root canal in TradACs cannot be achieved in ConsACs [62]. In the ConsAC group, possible contact of the plugger with the cavity walls may have limited orthograde access. With all of these considerations, no similar study in the literature allows a direct comparison to current findings on the impact of endodontic access cavity design on MTA apical plug porosity.

In TradACs, the highest open or total porosity was in the XPS, ultrasonic, and manual groups, respectively (*p* > 0.05). Additional techniques to manual condensation were ineffective in improving the quality of MTA obturation. Previous studies claimed that ultrasonic activation improves MTA obturation quality [26,34,35,44]. On the other hand, many studies have reported more dense [20], less porous [41], and more homogeneous [27] MTA obturation with manual condensation compared to ultrasonic activation. Kim et al. [63] and Adel et al. [43] reported that as the power of ultrasonic activation increases, leakage also increases, with the least leakage occurring in the manual technique. In the present study, the placement of MTA with 10 s of indirect ultrasonic activation resulted in higher porosity compared to manual placement alone in both the ConsAC and TradAC groups, consistent with the findings of El-Ma’aita et al. [20]. They noted higher percentages of porosity with indirect ultrasonic activation compared to manual condensation, and they recommended applying indirect ultrasonic activation for 5–10 s for optimal MTA obturation quality. In contrast, Parashos et al. [44] reported a higher incidence of radiographic voids in the manual technique, suggesting that 2–8 s of indirect ultrasonic activation resulted in better-quality MTA obturation compared to 10–18 s. Hence, the porosity level may increase with the duration of agitation [44], as it may lead to air trapping due to excessive vibrational force [63]. Despite applying indirect ultrasonic activation for the same duration (10 s) as El-Ma’aita et al. [20], Sisli et al. [34] reported an improvement in MTA obturation quality. The authors attributed this discrepancy in the studies to variations in MTA thickness. Consistent with the present study, Druktenis et al. [64] reported an increase in porosity percentages when direct ultrasonic activation was applied for 10 s on various bioceramic materials. In this study, the manual technique allows for more controlled condensation, regardless of cavity size. The nature of the manual technique, which inherently preserves tactile sensation, may contribute to this outcome. The aim of using the indirect ultrasonic technique was to observe whether ultrasonic activation increases the flow, settling, and compaction of MTA, evaluated through porosity percentages [32]. However, the additional application of ultrasonic or XPS after each manually applied layer seems to cause MTA particles to disperse or introduce air bubbles into the material. In TradACs, the ultrasonic technique resulted in more porosity compared to the manual technique. In ConsACs, it resulted in more porosity compared to XPS. This result may have arisen from the mixing procedures specified in the MTA manufacturer’s instructions, which are based on manual condensation [27]. In the broadest perspective, there exist inconsistent findings concerning the impact of indirect ultrasonic activation on porosity percentages in the literature. Nekoofar et al. [65] designed a custom-made device to ensure consistent manual condensation pressure for each test sample. They suggested that standardizing condensation pressure during MTA placement is crucial to avoid inconsistencies in study results. Additionally, the time of application and the temperature rise in the environment may have a more pronounced effect on the material particles than the method of ultrasonic energy application itself [65]. Non-identical experimental designs, variations in physical environmental conditions, and non-standardized manual compaction pressure may contribute to the divergent results observed in the literature between manual condensation and ultrasonic activation. 

In the ConsACs, total and open porosities were higher in the ultrasonic and manual groups compared to the TradACs. The highest open or total porosity was in the ultrasonic, XPS, and manual groups, respectively (*p* > 0.05). There are few studies evaluating the effectiveness of the rotary instruments for creating an MTA apical plug. The XPS file gradually transitions from the martensitic phase to the austenitic phase above 35 °C. Starting with initial dimensions of 0.27 mm and a 1% taper, it gradually reaches a final preparation size of 0.30 mm with a 4% taper. However, the XPS file system can adapt to the root canal anatomy and expand more in wider canals. In this study, the aim was to evaluate the potential effects of this characteristic movement to enhance MTA adaptation. An et al. [35] reported that MTA placed in the counterclockwise direction with a ProFile nickel–titanium file was more effective than manual placement. Researchers attributed this to the ProFile files, which remove debris in the coronal direction when rotating clockwise, facilitating apical obturation when working in the counterclockwise direction. The XPS used in the present study can perform three-dimensional mechanical preparation of the root canal without unnecessarily removing dentin tissue [66]. Similar to the ProFile, XPS has a design that allows the exit of dentin debris in the coronal direction [66]. However, in this study, unlike An et al. [35], the XPS group exhibited significantly higher open and total porosity, compared to the manual and ultrasonic groups in the TradACs. This may be because the serpentine rotation movement, albeit counterclockwise, somehow causes a more irregular obturation. Contrary to expectations, the serpentine motion of XPS, which enables its three-dimensional movement, resulted in a less dense obturation in TradACs compared to manual and ultrasonic techniques, instead of reducing the porosity of MTA. This may be due to the characteristic motion of XPS, which could have disrupted the fit of condensed MTA. However, in the XPS group, ConsACs showed less porosity compared to TradACs. The design and physical properties of any nickel–titanium file are developed to prepare the root canal system in the most effective and safe manner. Therefore, the experimental use of XPS for MTA condensation rather than its primary purpose may have resulted in it being less effective than the manual technique. Although XPS exhibited higher porosity compared to other techniques, it gave more successful results in open and total porosity in ConsACs. Moreover, XPS yielded more successful results than ultrasonic in terms of open and total porosity in ConsACs. The reason for this might be the enhanced MTA adaptation in ConsACs due to the finer instrument’s three-dimensional accessibility advantages. A smaller access cavity may have resulted in reduced oscillation and greater control of the snake-like movement of the XPS file. Ultrasonic in TradACs causing less porosity than XPS may have been attributed to the more efficient and controlled transmission of ultrasonic energy enabled by the TradAC design, facilitating the straight form. In studies conducted with extracted teeth, it is not possible to control dentin thickness. Although this study was conducted in a container with water at 37 °C, the stability of root canal temperature may not have been maintained under in vitro conditions [67]. Consequently, differences in dentin mass remaining according to cavity preparations could lead to variations in temperature, which in turn may result in different performances of XPS. 

In the TradACs, the percentage of closed porosity in the apical 2.5 mm region did not vary according to the application techniques. Additionally, the percentage of closed-apical porosity was lower in ConsACs than in TradACs in the manual application. Moreover, additional applications to manual technique have resulted in a decrease in closed coronal porosity. From this perspective, to explain the partial heterogeneous variations observed depending on the cavity design and application techniques in the porosities, further studies are needed.

Although there is no evidence to suggest that any root canal filling material or technique is most effective in treatment prognosis in endodontics, it is a well-known fact that the distance of the root canal filling to the apical foramen and the radiographic quality of the filling significantly influences treatment success [68]. While bacterial load control remains the most influential factor in the success of root canal treatment, the second significant factor is achieving a complete obturation extending to the apex [68]. Therefore, the presence of porosity in the MTA plug may impact the long-term treatment outcomes and tooth survival rates. The significant differences in porosity obtained in the current study may contribute to the clinician’s preferences during MTA apexification treatment. The findings of this study support the necessity to avoid ConsACs to create a denser apical plug, and manual condensation alone is sufficient. 

Within the limitations of this study, clinicians can ensure porosity control while establishing an apical plug using a traditional cavity design and manual techniques. Utilizing additional techniques or opting for minimal cavity design during apical plug formation may result in more porosity. This situation could jeopardize clinical success; therefore, standard approaches in MTA apical plug construction appear to be a safer option. On the other hand, in many teeth with open apices, endodontic treatment is required for caries-free, single-rooted, immature permanent teeth following a traumatic injury. In such teeth, clinicians may plan a more conservative cavity preparation to minimize hard tissue loss. In this situation, clinicians may need to be more careful to ensure effective adaptation of MTA. Nevertheless the results of this study cannot be directly used to predict the clinical prognosis of MTA apical plugs because the success of MTA apexification is dependent on numerous factors [30].

The limitations of this study include standardization bias due to the use of extracted human teeth. Although similar-sized and same-group teeth were selected for the current study, it is not possible to provide complete dimensional standardization of the samples. Another important limitation is that clinical conditions cannot be fully reflected in in vitro conditions. Among the other limitations of this study, there is the variability of human factors related to the hand mixing of MTA, which could lead to inconsistency in the mixture each time, despite meticulous adherence to the manufacturer’s instructions and the experiment being conducted by a single endodontist [69]. Additionally, no device was used to standardize manual condensation pressure, and anatomical matching was not performed when separating the experimental groups. The obturations were performed not on a phantom head but on plaster models; thus, dental ergonomic positions could not be mimicked. These situations should be taken into consideration when evaluating the findings of the study.

## 5. Conclusions

Within the limitations of this study, all samples exhibited different levels of porosity. No matter the cavity design, application techniques cause differences in porosity percentages. TradACs are more effective than ConsACs in controlling MTA porosity. Additional techniques applied alongside manual condensation were ineffective in reducing the open and total porosity of MTA, particularly in TradAC.

## Figures and Tables

**Table 1:** Inclusion and exclusion criteria for the teeth used in the study.

**Inclusion Criteria**
Upper central human teeth Single-rooted mature permanent teeth Minimum apical curvature Without any restorations 21 ± 2 mm inciso-apical length
**Exclusion Criteria**
Caries or fractures in crown or root Immature apices Internal or external root resorption Root canal calcification Oval canals

**Table 2:** The median and interquartile range (IQR) values of volume of porosity percentages (%) according to endodontic access cavity designs and MTA application techniques.

		Manual (*n* = 10)	Ultrasonic (*n* = 10)	XPS (*n* = 10)	(Manual-Ultrasonic) *p*	(Manual-XPS) *p*	(Ultrasonic-XPS) *p*
		Median (IQR)	Median (IQR)	Median (IQR)			
Closed-coronal	TradAC	0.08 (0.01–0.14)	0.00 (0.00–0.00)	0.00 (0.00–0.00)	**0.007**	**0.002**	0.303
	ConsAC	0.02 (0.01–0.03)	0.10 (0.04–0.15)	0.13 (0.04–0.39)	0.082	**0.019**	0.451
	*p*	0.256	**0.004**	**0.001**			
Closed-apical	TradAC	0.03 (0.01–0.04)	0.01 (0.00–0.03)	0.02 (0.00–0.03)	0.149	0.271	0.788
	ConsAC	0.01 (0.00–0.03)	0.05 (0.03–0.07)	0.06 (0.01–0.11)	0.029	**0.008**	0.649
	*p*	**0.023**	**0.015**	0.103			
Open-coronal	TradAC	8.4 (6.6–16.3)	27.2 (17.1–37.5)	36.8 (29.45–63.2)	**0.009**	**0.002**	**0.041**
	ConsAC	21.5 (17.2–26.3)	39.5 (35.2–48.7)	28.9 (25.6–32.5)	**0.007**	**0.041**	**0.023**
	*p*	**0.048**	**0.023**	0.112			
Open-apical	TradAC	11.7 (8.7–18.9)	23.9 (18.8–29.2)	47.4 (36.5–59.6)	**0.009**	**0.000**	**0.000**
	ConsAC	22.8 (18.0–27.7)	52.5 (40.6–61.0)	36.5 (26.6–44.1)	**0.005**	**0.019**	**0.049**
	*p*	**0.019**	**0.007**	**0.046**			
Total-coronal	TradAC	8.5 (6.7–16.3)	27.2 (17.1–37.6)	36.9 (29.4–63.2)	**0.009**	**0.002**	**0.041**
	ConsAC	21.5 (17.2–26.4)	39.6 (35.3–48.8)	29.0 (26–33.4)	**0.007**	**0.028**	**0.023**
	*p*	**0.045**	**0.023**	0.131			
Total-apical	TradAC	11.7 (8.8–19.0)	23.9 (18.8–29.3)	47.4 (36.5–59.6)	**0.009**	**0.000**	**0.000**
	ConsAC	22.8 (18.0–27.7)	52.5 (40.6–61.1)	36.6 (26.6–44.2)	**0.005**	**0.019**	**0.049**
	*p*	**0.019**	**0.007**	**0.049**			

Data were analyzed with Mann-Whitney *U* Test. For comparison of techniques between groups the Kruskal–Wallis and post hoc Bonferroni tests were used. XPS: XP-endo Shaper, ConsAC: Conservative access cavity, TradAC: Traditional access cavity. *p* < 0.05 is statistically significant.

## Data Availability

The data presented in this study are available from the corresponding author.
